# Supplementary material for: A TALE/HOX code unlocks WNT signalling response towards paraxial mesoderm
Source: Nat Commun. 2021 Aug 26;12:5136. doi: 10.1038/s41467-021-25370-4 (PMC8390530; doi:10.1038/s41467-021-25370-4)
Supplement: Supplementary file 1 — Supplementary Information [file 41467_2021_25370_MOESM1_ESM.pdf]

Supplementary Information

A TALE/HOX code unlocks WNT signalling response towards paraxial mesoderm

Luca Mariani, Xiaogang Guo, Niels Alvaro Menezes,
Anna Maria Drozd, Selgin Deniz Çakal, Qinhu Wang
and Elisabetta Ferretti

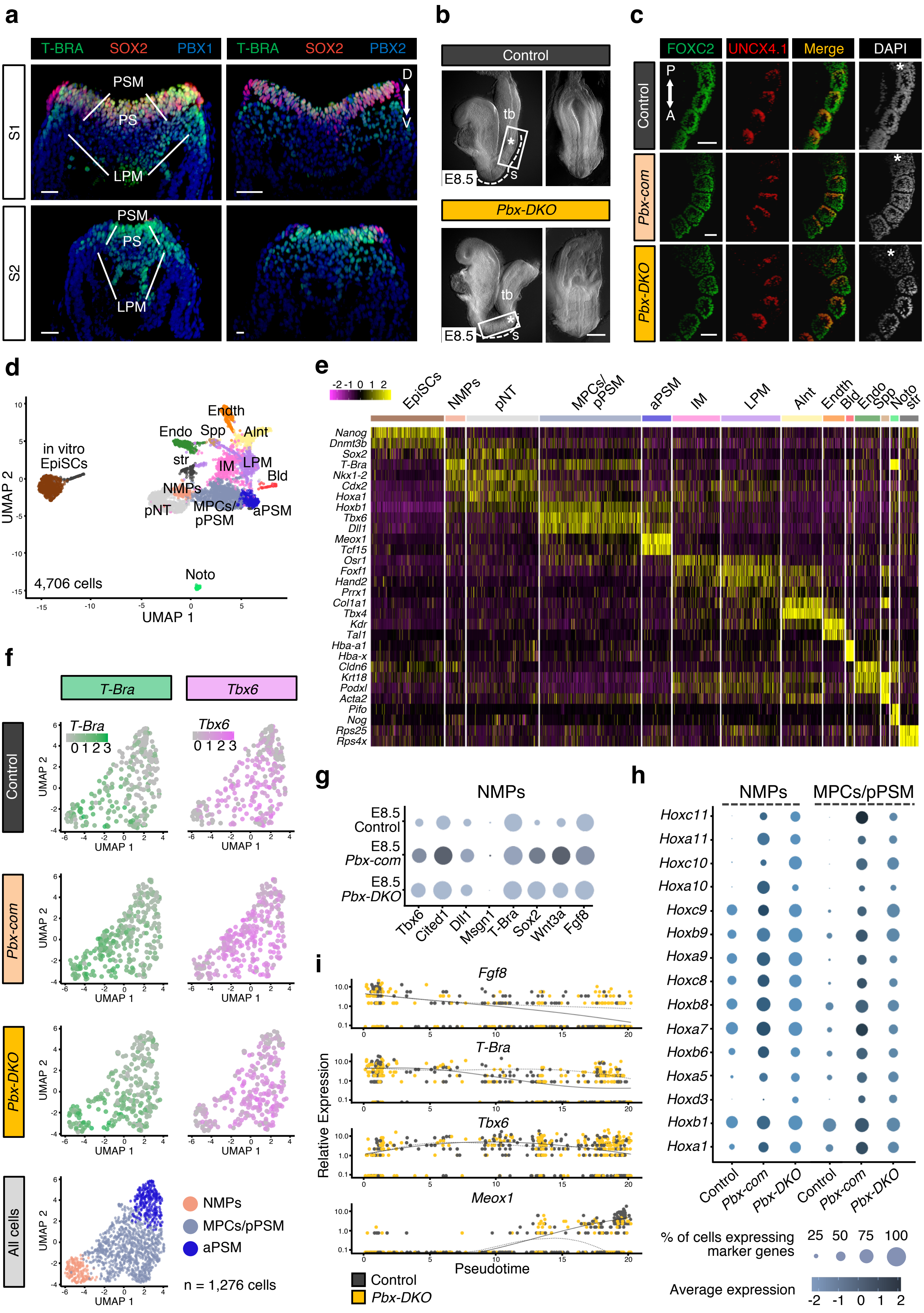
a
T-BRA SOX2 PBX1
T-BRA SOX2 PBX2
S1
S2
PSM
PS
LPM
D
V
b
Control
tb
s
E8.5
Pbx-DKO
c
FOXC2
UNCX4.1
Merge
DAPI
P
A
Control
Pbx-com
Pbx-DKO
d
UMAP 2
UMAP 1
in vitro EpiSCs
Endth
Endo
Spp
Alnt
str
IM
LPM
Bld
NMPs
pNT
MPCs/ pPSM
aPSM
Noto
4,706 cells
e
EpiSCs
NMPs
pNT
MPCs/ pPSM
aPSM
IM
LPM
Alnt
Endth
Bld
Endo
Spp
Noto
str
Nanog
Dnmt3b
Sox2
T-Bra
Nkx1-2
Cdx2
Hoxa1
Hoxb1
Tbx6
Dll1
Meox1
Tcf15
Osr1
Foxf1
Hand2
Prrx1
Col1a1
Tbx4
Kdr
Tal1
Hba-a1
Hba-x
Cldn6
Krt18
Podxl
Acta2
Pifo
Nog
Rps25
Rps4x
f
T-Bra
Tbx6
Control
Pbx-com
Pbx-DKO
All cells
NMPs
MPCs/pPSM
aPSM
n = 1,276 cells
g
NMPs
E8.5 Control
E8.5 Pbx-com
E8.5 Pbx-DKO
Tbx6
Cited1
Dll1
Msgn1
T-Bra
Sox2
Wnt3a
Fgf8
h
NMPs
MPCs/pPSM
Hoxc11
Hoxa11
Hoxc10
Hoxa10
Hoxc9
Hoxb9
Hoxa9
Hoxc8
Hoxb8
Hoxa7
Hoxb6
Hoxa5
Hoxd3
Hoxb1
Hoxa1
Control
Pbx-com
Pbx-DKO
% of cells expressing marker genes
25 50 75 100
Average expression
-2 -1 0 1 2
i
Fgf8
T-Bra
Tbx6
Meox1
Relative Expression
Pseudotime
Control
Pbx-DKO

Supplementary Fig. 1 I PBX play a critical role in the initial acquisition of PM fate. **a**, Immunofluorescence staining of E8.5 mouse tailbuds shows PBX1 and PBX2 localisation in the primitive streak (PS), pre-somitic mesoderm (PSM) and lateral plate mesoderm (LPM). Two transverse sections through the caudal lateral epiblast are shown. D dorsal, V ventral. Scale bars: 20 μm. **b**, Gross morphology of E8.5 *Pbx-DKO* embryos showing enlarged tailbud (tb), shortened trunk and rudimentary somites (s). Lateral view on the left and posterior view on the right. The white frames highlight the regions shown in **c**. Scale bar: 200 μm. **c**, Representative immunofluorescence staining for FOXC2 (green) and UNCX4.1 (red) on sagittal sections of E8.5 *Pbx-com* and *Pbx-DKO* embryos, displaying defective posterior somites development. P posterior, A anterior. Scale bars: 50 μm. In **b** and **c**, asterisks denote the newly generated somites. **d**, ScRNA-seq of E8.5/E9.0 control, *Pbx-com* and *Pbx-DKO* embryonic tailbuds. Unsupervised clustering and UMAP visualisation of embryonic cells together with spiked-in EpiSCs support efficient removal of batch-effect. Individual cells are coloured according to annotated cluster identities: EpiSCs epiblast stem cells, NMPs neuromesodermal progenitors, pNT pre-neural tube, MPCs/pPSM mesodermal progenitor cells/posterior pre-somitic mesoderm, aPSM anterior pre-somitic mesoderm, IM intermediate mesoderm, LPM lateral plate mesoderm, Alnt allantois, Endth Endothelial cells, Bld blood, Endo endoderm, Spp splanchnopleura, Noto notochord, str stressed cells. **e**, Heatmap showing key genes distinguishing 14 clusters in E8.5/E9.0 tailbuds and in vitro EpiSCs. Colour bar indicates the intensity associated with normalised expression values. **f**, UMAP of control, *Pbx-com* and *Pbx-DKO* cells in NMPs (light pink), MPCs/pPSM (light blue) and aPSM (dark blue) clusters. Cells are coloured by expression of the NMP marker *T-Bra* (green) and the PSM marker *Tbx6* (magenta), showing enrichment of $T\text{-}Bra^{pos}$;$Tbx6^{pos}$ MPCs in E8.5 *Pbx* mutants compared to controls. Colour bars indicate the intensity associated with normalised expression values. **g**, Expression analysis of selected lineage markers within the NMPs cluster isolated from E8.5 control and *Pbx* mutant tailbuds. Dot plot shows the relative expression of the NMP markers *T-Bra*, *Sox2*, *Wnt3a*, *Fgf8*, and the mesodermal genes *Tbx6*, *Cited1*, *Dll1*, *Msgn1*, indicating co-expression of NMP and MPC markers in *Pbx-com* and *Pbx-DKO* embryos. **h**, Expression analysis of *Hox* genes within the NMPs and MPCs/pPSM clusters isolated from E8.5 control and *Pbx* mutant tailbuds. Dot plot shows enhanced expression of 5' *Hox* genes in *Pbx-com* and *Pbx-DKO* embryos. Colour bar indicates the intensity associated with normalised expression values. **i**, Monocle's pseudotemporal ordering of NMPs along a path towards aPSM, showing the distribution of NMP markers *Fgf8* and *T-Bra*, and PSM genes *Tbx6* and *Meox1* in control (black) and *Pbx-DKO* cells (yellow). The graphs highlight the inability of *Pbx-DKO* cells to reach a mature aPSM stage, resulting in their accumulation as early progenitors.

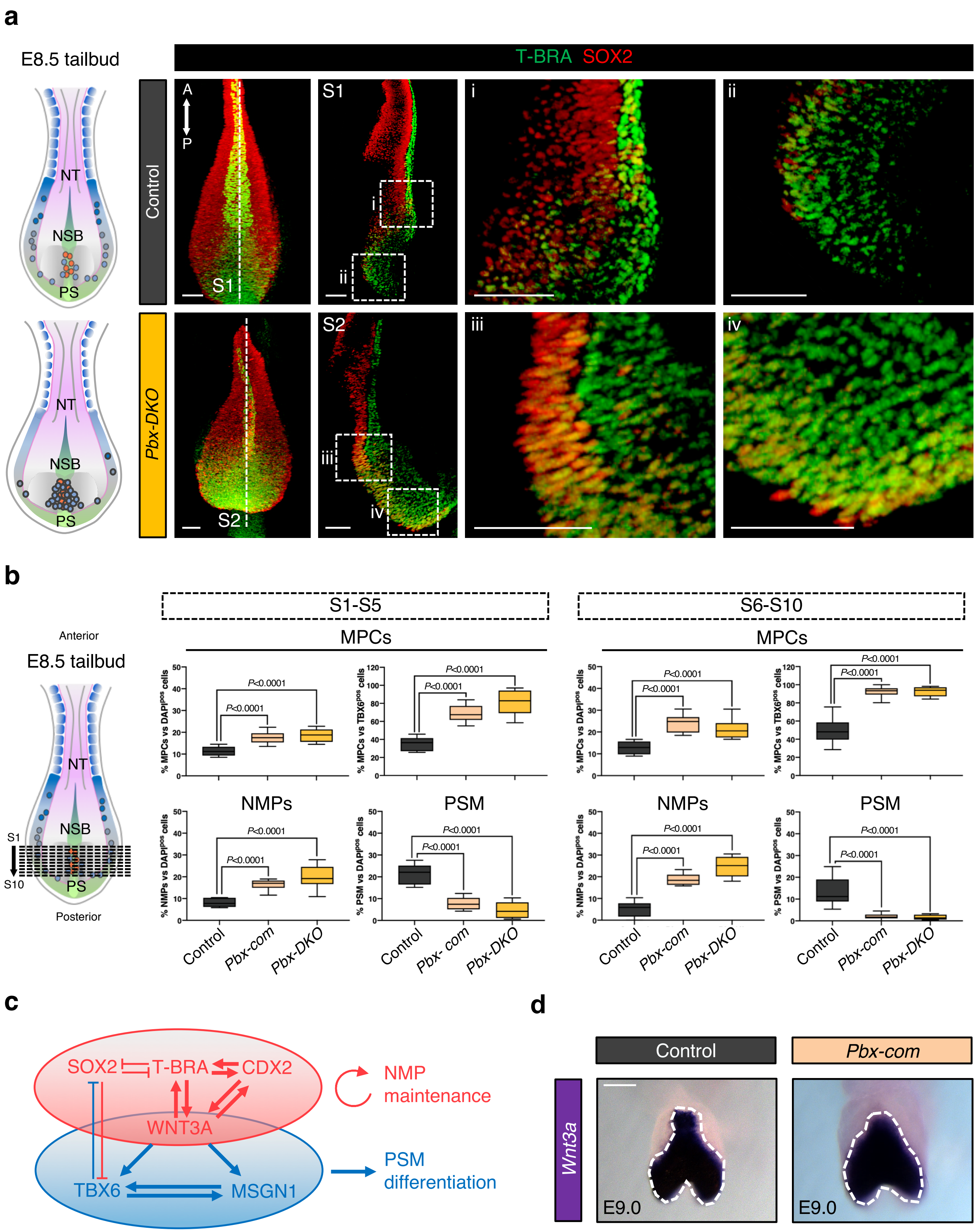
a
E8.5 tailbud
T-BRA SOX2
Control
Pbx-DKO
NT
NSB
PS
A
P
S1
S2
i
ii
iii
iv
b
S1-S5
S6-S10
Anterior
E8.5 tailbud
Posterior
MPCs
NMPs
PSM
P<0.0001
Control
Pbx-com
Pbx-DKO
c
SOX2
T-BRA
CDX2
WNT3A
TBX6
MSGN1
NMP maintenance
PSM differentiation
d
Control
Pbx-com
Wnt3a
E9.0

Supplementary Fig. 2 | Impaired PSM formation in *Pbx* mutant embryos. a, Confocal maximum intensity projection 3D reconstruction of E8.5 control and *Pbx-DKO* tailbuds stained with T-BRA (green) and SOX2 (red). Sagittal sections, S1 and S2, along the embryonic midline show accumulation of T-BRA^{pos};SOX2^{pos} NMPs in CLE and posterior tailbud of *Pbx-DKO* (higher magnification shown in panels iii and iv) compared to controls (higher magnification shown in panels i and ii). The cartoons on the left depict E8.5 control and *Pbx-DKO* tailbuds, with NMPs represented as red dots and MPCs as blue dots. Scale bars: 50 μm. **b**, Box plots assessing the proportion of T-BRA^{pos};TBX6^{pos} cells (MPCs) relative to DAPI^{pos} cells (total cells) or to TBX6^{pos} cells (PSM), and T-BRA^{pos};SOX2^{pos} cells (NMPs) and TBX6^{pos} cells (PSM) relative to DAPI^{pos} cells. Black dashed lines in the cartoon indicate the plane of sections. Sections S1-S5 represent the anterior CLE, while sections S6-S10 are relative to the posterior CLE and show the robust accumulation of NMPs and MPCs in the posterior tailbud. The box contains the 25th to 75th percentiles of the dataset, the centre line denotes the median value (50th percentile) and the whiskers extend from the smallest to the largest value. *P*-values were calculated by one-way ANOVA and Tukey's test for multiple comparisons and are indicated in the figure. In **a** and **b**: NSB node-streak border, PS primitive streak, NT neural tube, A anterior, P posterior. **c**, Schematics of the regulatory network operating in the mouse tailbud with WNT signalling sustaining both maintenance of the NMP state (red) and PSM differentiation (blue). **d**, *Wnt3a* mRNA *in situ* hybridization analysis of E9.0 tailbuds reveals expanded *Wnt3a* expression in the progenitor zone (delimited by white dashed lines) of *Pbx-com* embryos (light pink) compared to controls (black). Scale bar: 100 μm.

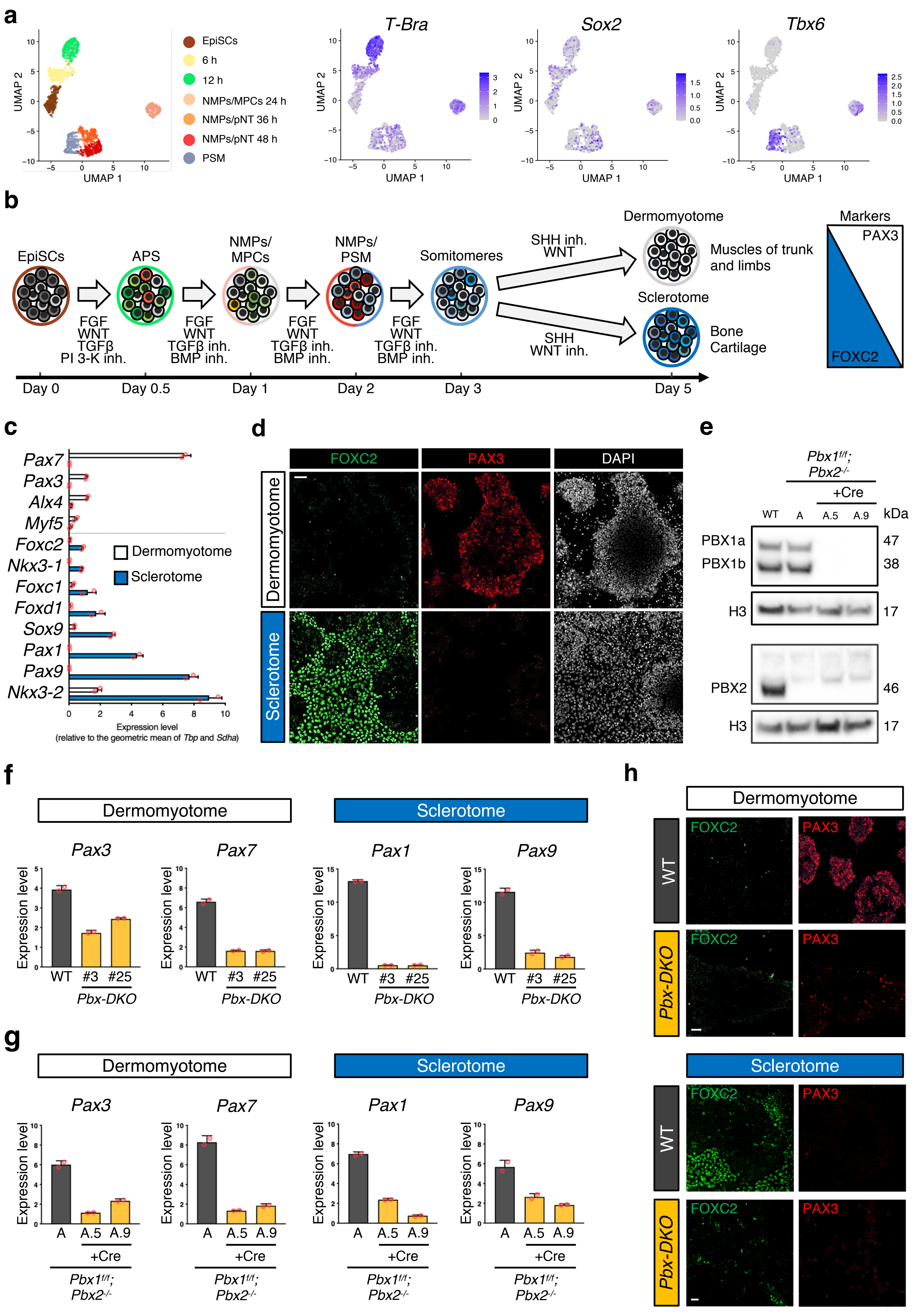
a
EpiSCs
6 h
12 h
NMPs/MPCs 24 h
NMPs/pNT 36 h
NMPs/pNT 48 h
PSM
UMAP 1
UMAP 2
T-Bra
Sox2
Tbx6
b
EpiSCs
APS
NMPs/ MPCs
NMPs/ PSM
Somitomeres
Dermomyotome
Sclerotome
Muscles of trunk and limbs
Bone Cartilage
Markers
PAX3
FOXC2
FGF WNT TGFβ PI 3-K inh.
FGF WNT TGFβ inh. BMP inh.
SHH inh. WNT
SHH WNT inh.
Day 0
Day 0.5
Day 1
Day 2
Day 3
Day 5
c
Pax7
Pax3
Alx4
Myf5
Foxc2
Nkx3-1
Foxc1
Foxd1
Sox9
Pax1
Pax9
Nkx3-2
Dermomyotome
Sclerotome
Expression level
(relative to the geometric mean of Tbp and Sdha)
d
FOXC2
PAX3
DAPI
Dermomyotome
Sclerotome
e
Pbx1f/f; Pbx2-/-
+Cre
WT
A
A.5
A.9
kDa
PBX1a
PBX1b
H3
PBX2
47
38
17
46
17
f
Dermomyotome
Sclerotome
Pax3
Pax7
Pax1
Pax9
Expression level
WT
#3
#25
Pbx-DKO
g
Dermomyotome
Sclerotome
Pax3
Pax7
Pax1
Pax9
Expression level
A
A.5
A.9
+Cre
Pbx1f/f; Pbx2-/-
h
Dermomyotome
Sclerotome
WT
Pbx-DKO
FOXC2
PAX3

Supplementary Fig. 3 I In vitro differentiation of WT and *Pbx-DKO* cells towards somite-derived tissues recapitulates the phenotype of *Pbx* mutant embryos. a, UMAP visualization of in vitro PSM differentiation. Individual cells are coloured by expression of *T-Bra*, *Sox2* and *Tbx6*. All cells express *T-Bra* after 12 h of PS induction, indicating the high efficiency of the differentiation protocol. Colour bars indicate the intensity associated with normalised expression values. **b**, Schematics of EpiSC differentiation towards anterior primitive streak (APS), pre-somitic mesoderm (PSM) and somite-derived tissues, generated by providing either Sonic Hedgehog (SHH) or WNT to mirror the formation of sclerotome and dermomyotome, respectively. **c**, Relative mRNA expression of dermomyotome (white) and sclerotome (blue) genes measured by RT-qPCR at day 5 of differentiation in WT cells. **d**, Representative immunofluorescence staining for the sclerotome marker FOXC2 (green) and the dermomyotome marker PAX3 (red) at day 5 of differentiation, showing the differentiation potential of the NMPs generated in vitro. Scale bar: 50 μm. **e**, Western blot analysis confirming the absence of PBX1 and PBX2 proteins in the *Pbx-DKO* lines generated by CRE-mediated recombination of $Pbx1^{flox/flox}$;$Pbx2^{-/-}$ Bl6-derived EpiSCs. **f-g**, Relative mRNA expression of dermomyotome (white) and sclerotome (blue) genes measured by RT-qPCR at day 5 of differentiation in WT (black) and *Pbx-DKO* lines (yellow) generated by CRISPR/Cas9 (**f**) or CRE-mediated recombination (**g**). **h**, Representative immunofluorescence staining for the signature markers FOXC2 (green, sclerotome) and PAX3 (red, dermomyotome) at day 5 of differentiation, showing impaired ability of the *Pbx-DKO* lines to differentiate into dermomyotome and sclerotome, mimicking the defects of the *Pbx* mutant embryos. Scale bars: 50 μm. Data are mean ± s.e.m. (n=2 biological replicates) in **c**, **f** and **g**.

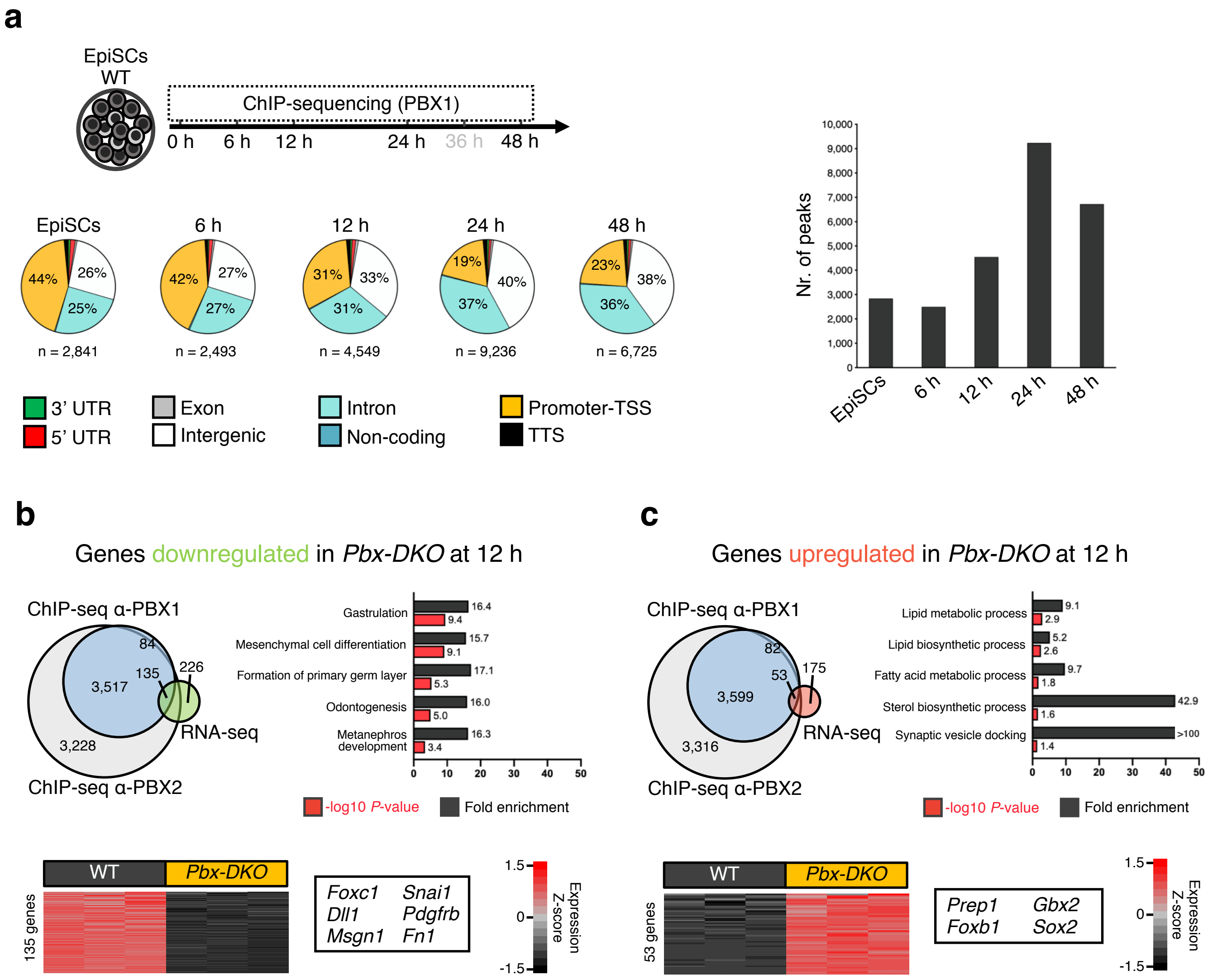

Supplementary Fig. 4 I PBX proteins are dynamically recruited to PM regulatory regions during differentiation. a, Left: annotation of the PBX1 ChIP-seq peaks according to their genomic position. The regions bound by PBX1 are subdivided based on binding occurrence at transcription start sites (TSSs) (-1 kb to +100 bp), 5' UTRs (-10 kb to -1 kb), transcription termination sites (TTSs) (-100 bp to +1 kb), 3' UTRs (+1 kb to +10 kb), introns, exons and intergenic regions; n = ChIP-seq peaks. Right: histogram showing the number of PBX1 ChIP-seq peaks across differentiation. **b,** Top: overlap of downregulated genes in *Pbx-DKO* cells (RNA-seq) and genes associated with PBX1 and PBX2-occupied regions at 12 h (ChIP-seq). GO-term enrichment analysis shows correlation with terms associated with general mesoderm and PSM differentiation (Binomial test with Bonferroni correction). Bottom: heatmap displaying RNA-seq expression Z-scores computed for 135 downregulated genes in *Pbx-DKO* (yellow) compared to WT cells (black). Representative markers are indicated. **c**, Top: overlap of upregulated genes in *Pbx-DKO* cells (RNA-seq) and genes associated with PBX1 and PBX2-occupied regions at 12 h (ChIP-seq). GO-term enrichment analysis shows overrepresentation of terms associated with metabolic processes (Binomial test with Bonferroni correction). Bottom: heatmap showing RNA-seq expression Z-scores computed for 53 upregulated genes in *Pbx-DKO* (yellow) compared to WT cells (black). Representative markers are indicated. In **b** and **c**, significance was assessed by DEseq2 on the basis of two-sided Wald test with Benjamini–Hochberg adjusted *P*-values ($P \leq 0.05$, fold-change ≥ 1.5). n=3 biological replicates.

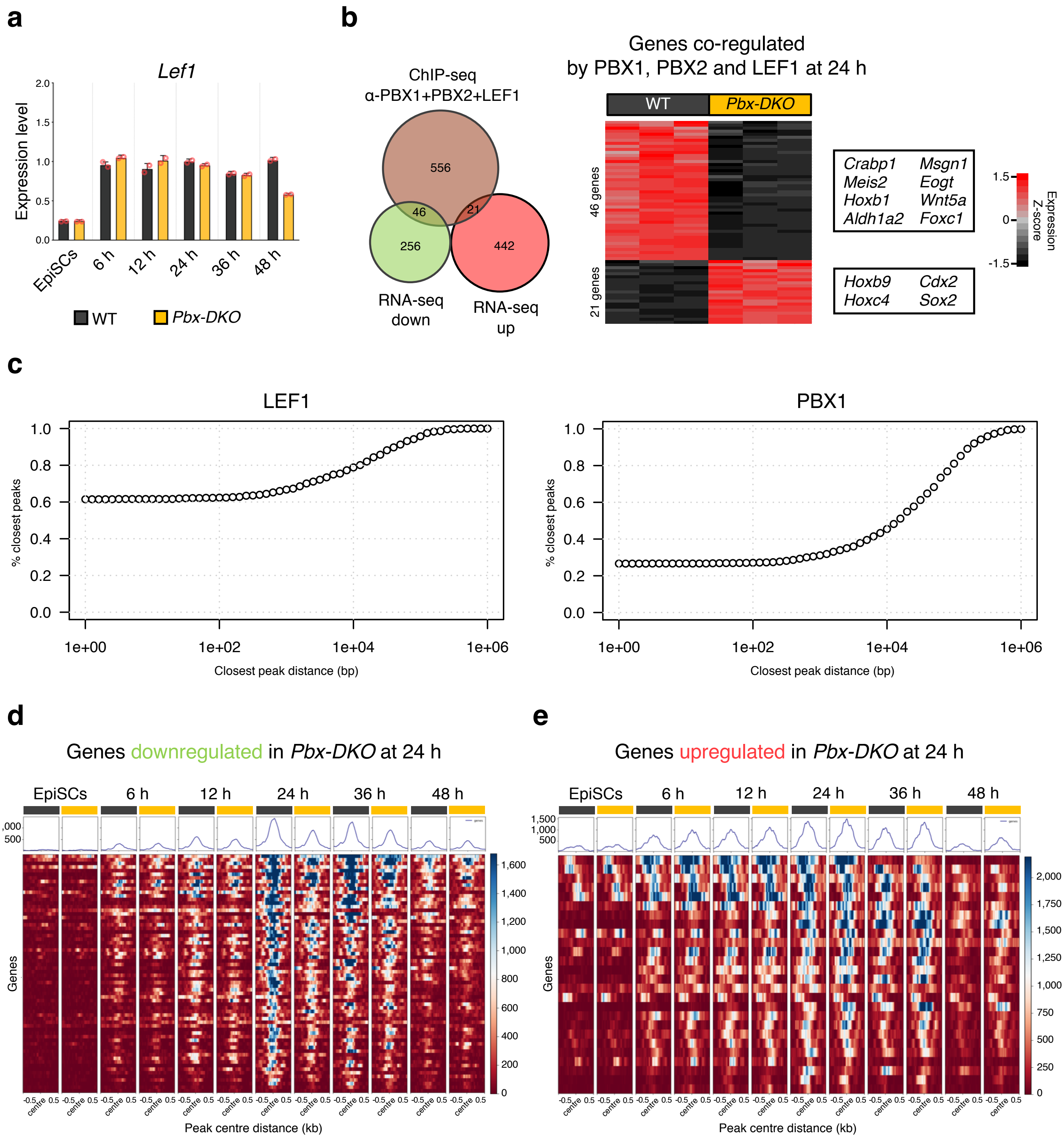
a
Lef1
Expression level
2.0
1.5
1.0
0.5
0.0
EpiSCs
6 h
12 h
24 h
36 h
48 h
WT
Pbx-DKO
b
ChIP-seq
α-PBX1+PBX2+LEF1
556
46
21
256
442
RNA-seq down
RNA-seq up
Genes co-regulated by PBX1, PBX2 and LEF1 at 24 h
WT
Pbx-DKO
46 genes
21 genes
Crabp1
Msgn1
Meis2
Eogt
Hoxb1
Wnt5a
Aldh1a2
Foxc1
Hoxb9
Cdx2
Hoxc4
Sox2
1.5
0
-1.5
Expression Z-score
c
LEF1
PBX1
% closest peaks
1.0
0.8
0.6
0.4
0.2
0.0
1e+00
1e+02
1e+04
1e+06
Closest peak distance (bp)
d
Genes downregulated in Pbx-DKO at 24 h
e
Genes upregulated in Pbx-DKO at 24 h
EpiSCs
6 h
12 h
24 h
36 h
48 h
1,500
1,000
500
Genes
1,600
1,400
1,200
1,000
800
600
400
200
0
2,000
1,750
1,500
1,250
1,000
750
500
250
0
-0.5
centre
0.5
Peak centre distance (kb)

Supplementary Fig. 5 l Recruitment of PBX-LEF1 on PM genes. a, Relative mRNA expression of *Lef1* measured by RT-qPCR, exhibiting comparable expression in WT (black) and *Pbx-DKO* cells (yellow) along PSM differentiation. Data are mean ± s.e.m. n=2 biological replicates. **b**, Left: overlap of genes co-occupied by PBX1, PBX2 and LEF1 (brown) with the downregulated (green) and upregulated (red) genes in *Pbx-DKO* cells compared to WT, showing a total of 67 genes under the direct control of combined PBX-LEF1 activity. Right: heatmap displaying RNA-seq expression Z-scores computed for PBX-LEF1 co-regulated genes that are differentially expressed in WT and *Pbx-DKO* cells at 24 h ($P \leq 0.05$, fold-change ≥ 1.5). Representative subsets of target genes directly regulated by PBX and LEF1 are reported in the boxes and include important PSM regulators. n=3 biological replicates. Significance was assessed by DEseq2 on the basis of two-sided Wald test with Benjamini–Hochberg adjusted *P*-values. **c**, Histograms showing the distribution of distances between LEF1 and PBX1 ChIP-seq peaks. Left: for each LEF1 peak identified at 24 h of differentiation, bedtools was used to determine the closest PBX1 peaks; right: for each PBX1 peak identified at 24 h of differentiation, bedtools was used to determine the closest LEF1 peaks. **d**, Heatmap of chromatin accessibility of WT (black) and *Pbx-DKO* cells (yellow) at LEF1-occupied regions that are associated with downregulated PBX-LEF1 target genes in *Pbx-DKO* along PSM differentiation, showing reduced accessibility in *Pbx-DKO* cells at 24 and 36 h*.* **e**, Heatmap of chromatin accessibility at LEF1-bound regions associated with upregulated PBX-LEF1 target genes in *Pbx-DKO* (yellow) compared to WT (black) cells. Chromatin accessibility is comparable between WT and *Pbx-DKO* cells. The scale represents normalised counts (RPKM) for ATAC-seq peak signals ± 500 bp around the centre of the peak in **d** and **e**.

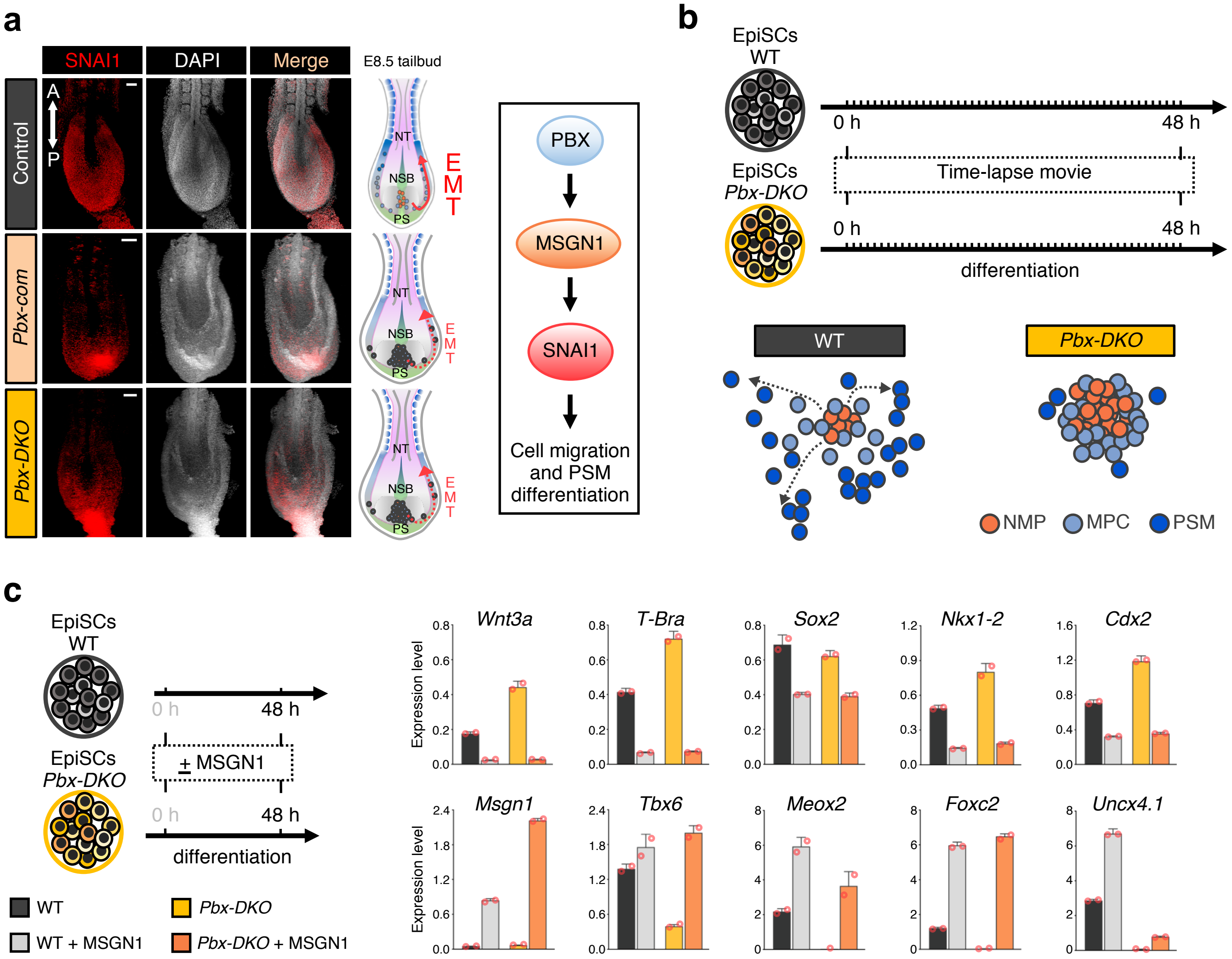

Supplementary Fig. 6 I PBX control *Msgn1* expression and in turn EMT and migration of MPCs. a, Confocal maximum intensity projection images of E8.5 control, *Pbx-com* and *Pbx-DKO* embryos probed with SNAI1 antibody (red) and counterstained with DAPI (grey), showing downregulation of the MSGN1 target and EMT marker SNAI1 in the tailbud of *Pbx* mutants. The cartoons on the right depict E8.5 control and *Pbx* mutant tailbuds, with NMPs represented as red dots and MPCs as blue dots. NSB node-streak border, PS primitive streak, NT neural tube, A anterior, P posterior, EMT epithelial-to-mesenchymal transition. Scale bars: 50 μm. The gene regulatory network involving PBX, MSGN1 and SNAI1 is reported in the box. **b**, Top: schematics of EpiSC differentiation to PSM. Pictures were acquired every hour to make time-lapse videos of in vitro differentiation (see Supplementary Movies 1, 2). Bottom: cartoon illustrating the migratory behaviour of WT and *Pbx-DKO* cells upon differentiation. WT cells progressively leave the NMP zone, while mutant cells fail to migrate and accumulate in the progenitor area. **c**, Rescue of the *Pbx* mutant paraxial mesoderm phenotype by lentivirus-mediated expression of *Msgn1* in *Pbx-DKO* cells (orange), resulting in downregulation of NMP markers (*Wnt3a, T-Bra*, *Sox2*, *Nkx1-2* and *Cdx2*) and upregulation of PSM genes (*Msgn1, Tbx6*, *Meox2*, *Foxc2* and *Uncx4.1*). Data are mean ± s.e.m. n=2 biological replicates.

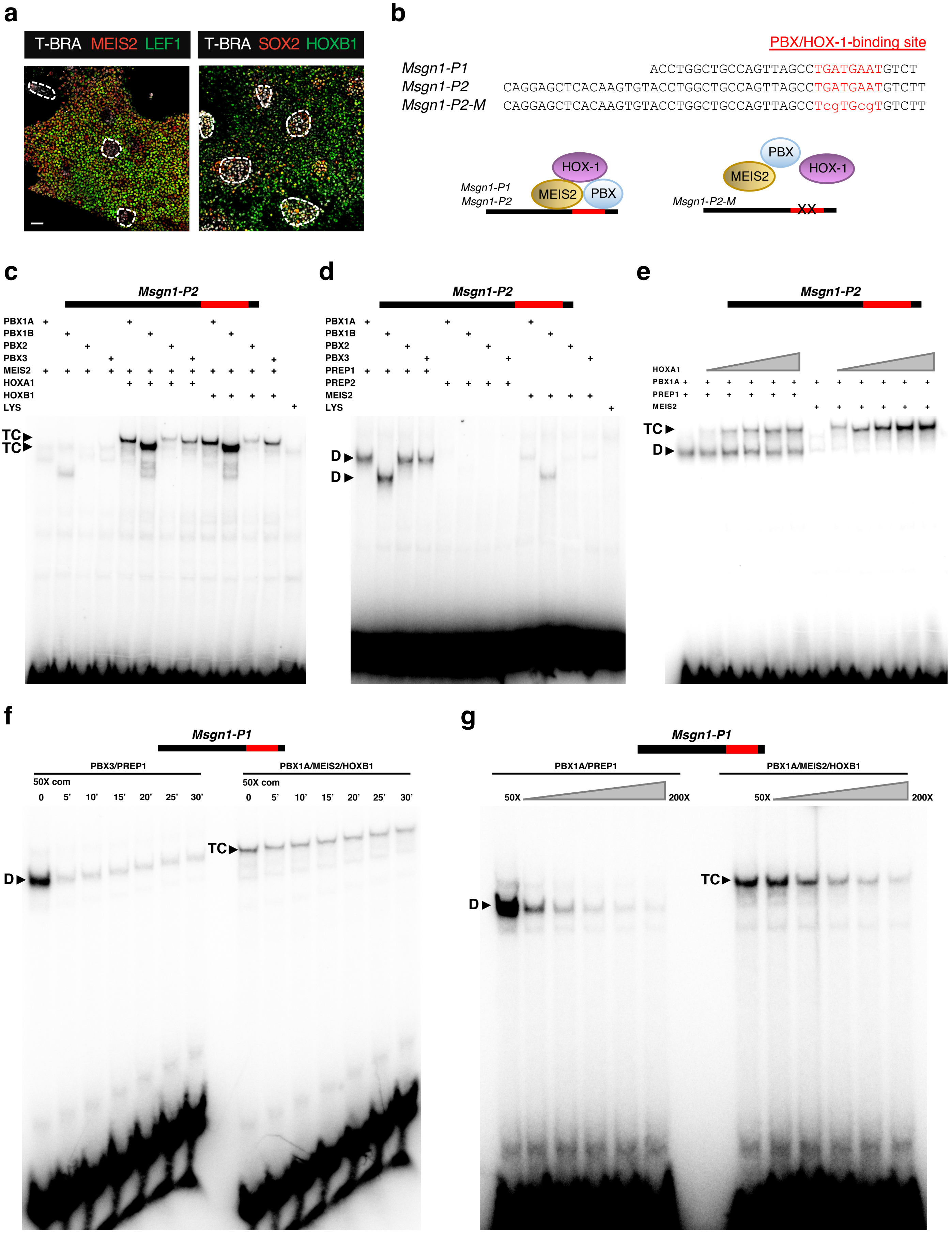
a
T-BRA MEIS2 LEF1
T-BRA SOX2 HOXB1
b
PBX/HOX-1-binding site
Msgn1-P1 ACCTGGCTGCCAGTTAGCCTGATGAATGTCT
Msgn1-P2 CAGGAGCTCACAAGTGTACCTGGCTGCCAGTTAGCCTGATGAATGTCTT
Msgn1-P2-M CAGGAGCTCACAAGTGTACCTGGCTGCCAGTTAGCCTcgTGcgTGTCTT
HOX-1
MEIS2
PBX
Msgn1-P1
Msgn1-P2
Msgn1-P2-M
XX
c
Msgn1-P2
PBX1A
PBX1B
PBX2
PBX3
MEIS2
HOXA1
HOXB1
LYS
TC
TC
d
Msgn1-P2
PBX1A
PBX1B
PBX2
PBX3
PREP1
PREP2
MEIS2
LYS
D
D
e
Msgn1-P2
HOXA1
PBX1A
PREP1
MEIS2
TC
D
f
Msgn1-P1
PBX3/PREP1
PBX1A/MEIS2/HOXB1
50X com
0 5' 10' 15' 20' 25' 30'
50X com
0 5' 10' 15' 20' 25' 30'
D
TC
g
Msgn1-P1
PBX1A/PREP1
PBX1A/MEIS2/HOXB1
50X 200X
50X 200X
D
TC

Supplementary Fig. 7 l Assembly of a specific PBX/MEIS2/HOX-1 ternary complex on *Msgn1* promoter. a, Representative immunofluorescence staining for T-BRA (white), MEIS2 (red) and LEF1 (green) (left), and T-BRA (white), SOX2 (red) and HOXB1 (green) (right), at 48 h of differentiation in WT cells. MEIS2, HOXB1 and LEF1 are enriched in cells adopting a PSM identity, in agreement with their suggested role in driving PSM differentiation from NMPs (T-BRA^{pos};SOX2^{pos}, dashed white lines). Scale bar: 50 μm. **b**, Schematic representation of all oligonucleotides employed in the EMSA experiments and formation of the PBX/MEIS2/HOXA1 complex. The PBX/HOX-1-binding site is highlighted in red. **c**, EMSA with in vitro-translated PBX1A, PBX1B, PBX2, PBX3, MEIS2, HOXA1 and HOXB1 proteins, showing that a ternary complex (TC) is formed with any combination of TALE and HOX-1 proteins and the *Msgn1-P2* oligonucleotide. The composition of each binding reaction is indicated at the top. LYS indicates the endogenous activity present in the reticulocyte lysate. **d**, In the absence of HOX-1, only PBX/PREP1 heterodimeric complexes (D) can bind to *Msgn1-P2* oligonucleotide by EMSA. **e**, EMSA with increased levels of in vitro-translated HOXA1, showing that the ternary complexes PBX/MEIS2/HOXA1 (TC) have higher affinity for *Msgn1-P2* compared to the PBX/PREP1 complexes, which bind also as heterodimers (D). **f-g**, Binding affinity properties of the ternary complex tested by EMSA. **f**, Analysis of dissociation rate of the complexes formed on *Msgn1-P1.* The oligo was incubated on ice for 10 minutes with the in vitro-translated proteins, as indicated at the top. A 50X-fold excess of unlabelled *Msgn1-P1* was added and aliquots analysed by EMSA every 5 min as indicated. The ternary complex PBX1A/MEIS2/HOXB1 (TC) is more stable and dissociates slowly compared to the dimeric complex PBX3/PREP1 (D). **g**, EMSA competition assays. Dimeric complex PBX1A/PREP1 (D), ternary complex PBX1A/MEIS2/HOXB1 (TC) and labelled *Msgn1-P1* were incubated with increased amount of cold *Msgn1-P1* competitor, showing higher affinity of the TC for *Msgn1-P1* oligonucleotide.

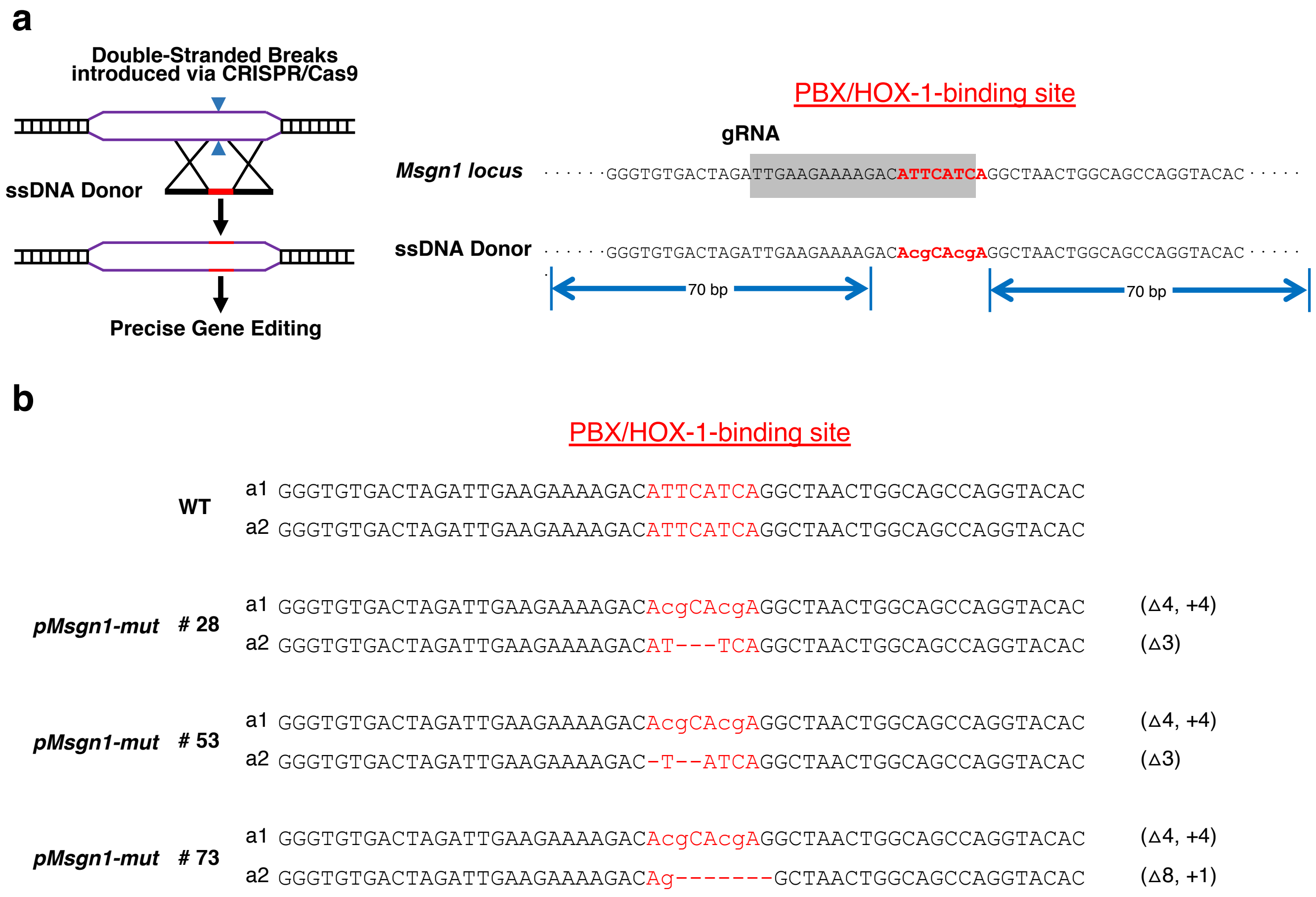

Supplementary Fig. 8 I Strategy to generate EpiSC lines carrying point mutations abrogating recruitment of the PBX complexes to the *Msgn1* regulatory region. a, Left: CRISPR/Cas9-based strategy used to mutagenise the PBX/HOX-1-binding site on *Msgn1* promoter. Right, top: guide RNA (gRNA) sequence (in grey) and PBX/HOX-1-binding site (in red). Right, bottom: single-stranded DNA (ssDNA) with 70 bp *Msgn1* homology arms flanking the mutagenised PBX/HOX-1-binding site, used for precise CRISPR/Cas9 editing and homology-directed repair (HDR). Lowercase letters in red indicate the introduced mutations. **b**, DNA sequencing readouts of the three independently generated *pMsgn1-mut* lines used in this study, revealing precise editing of one allele (a1) and introduction of small indels in the other allele (a2). The wild-type sequence is shown at the top with the PBX/HOX-1-binding site labelled in red. Red dashes indicate deletions and lowercase letters insertions. Number of deleted (Δ) or inserted (+) base pairs is reported in brackets. Of note, recruitment of the PBX/HOX-1 complex to the PBX/HOX-1-binding site is abolished in all three lines, as representatively shown in Fig. 7d.

Supplementary Table 1. Summary of cytokines and signalling pathway inhibitors used in this study.

Item name	Description	Company	Catalog nr.
A-83-01	TGF-β inhibitor	Tocris	2939
Activin A	TGF-β agonist	Peprotech	120-14E
C59	WNT inhibitor	Cellagen Technology	C7641
CHIR-99021	WNT agonist	Axon MedChem	1386
bFGF	FGF agonist	Peprotech	450-33
GDC-0449	Hedgehog inhibitor	Axon MedChem	1500
LDN193189	BMP inhibitor	Sigma	SML0559
PIK-90	PI 3-K inhibitor	EMD Millipore	528117
SAG 21k	Hedgehog agonist	Tocris	5282
XAV939	WNT inhibitor	Sigma	X3004

Supplementary Table 2. Antibodies used for immunofluorescence staining of adherent cells.

**Primary Antibody (clonality, host)**	**Company**	**Catalog nr.**	**Dilution**
FOXC2 (polyclonal, Sheep)	R&D Systems	AF6989	1:100
HOXB1 (polyclonal, Sheep)	R&D Systems	AF6318	1:100
LEF1 (monoclonal, Rabbit)	Cell Signaling Technology	2230	1:100
MEIS2 (monoclonal, Mouse)	Santa Cruz Biotechnology	sc81986	1:100
PAX3 (monoclonal, Mouse)	DSHB	AB_528426	1:100
SOX2 (monoclonal, Mouse)	Santa Cruz Biotechnology	sc-365823	1:100
T-BRA (polyclonal, Goat)	R&D Systems	AF2085	1:100
T-BRA (monoclonal, Rabbit)	R&D Systems	MAB20851	1:100
TBX6 (polyclonal, Goat)	R&D Systems	AF4744	1:50
**Secondary Antibody (host)**	**Company**	**Catalog nr.**	**Dilution**
Goat Alexa Fluor 568 (Donkey)	Thermo Fisher Scientific	A-11057	1:200
Mouse Alexa Fluor 647 (Donkey)	Thermo Fisher Scientific	A-31571	1:200
Rabbit Alexa Fluor 488 (Donkey)	Thermo Fisher Scientific	A-21206	1:200
Sheep Alexa Fluor 488 (Donkey)	Thermo Fisher Scientific	A-11015	1:200

Supplementary Table 3. Antibodies used for immunofluorescence staining of embryos.

**Primary Antibody (clonality, host)**	**Company**	**Catalog nr.**	**Dilution**
FOXC2 (polyclonal, Sheep)	R&D Systems	AF6989	1:100
LEF1 (monoclonal, Rabbit)	Cell Signaling Technology	2230	1:100
PBX1 (polyclonal, Rabbit)	Cell Signaling Technology	4342	1:100
PBX2 (polyclonal, Rabbit)	Thermo Fisher Scientific	PA5-63964	1:100
SNAI1 (monoclonal, Rabbit)	Cell Signaling Technology	3879	1:100
SOX2 (monoclonal, Mouse)	Santa Cruz Biotechnology	sc-365823	1:100
T-BRA (polyclonal, Goat)	R&D Systems	AF2085	1:100
T-BRA (monoclonal, Rabbit)	R&D Systems	MAB20851	1:100
TBX6 (polyclonal, Goat)	R&D Systems	AF4744	1:50
UNCX (polyclonal, Rabbit)	Thermo Fisher Scientific	PA5-66568	1:100
**Secondary Antibody (host)**	**Company**	**Catalog nr.**	**Dilution**
Goat Alexa Fluor 568 (Donkey)	Thermo Fisher Scientific	A-11057	1:500
Mouse Alexa Fluor 647 (Donkey)	Thermo Fisher Scientific	A-31571	1:500
Rabbit Alexa Fluor 488 (Donkey)	Thermo Fisher Scientific	A-21206	1:500
Sheep Alexa Fluor 488 (Donkey)	Thermo Fisher Scientific	A-11015	1:500

Supplementary Table 4. Antibodies used for ChIP.

Primary Antibody (clonality, host)	Company	Catalog nr.	Dilution
IgG (polyclonal, Goat)	R&D Systems	AB-108-C	1:1,000
IgG (polyclonal, Rabbit)	Cell Signaling Technology	2729	1:1,000
LEF1 (monoclonal, Rabbit)	Cell Signaling Technology	2230	1:100
MEIS2 (polyclonal, Rabbit)	Abcam	ab73164	1:125
PBX1 (polyclonal, Rabbit)	Cell Signaling Technology	4342	1:100
PBX2 (polyclonal, Rabbit)	Thermo Fisher Scientific	PA5-63964	1:125
PREP1 (polyclonal, Rabbit)	Thermo Fisher Scientific	PA5-30244	1:125
T-BRA (polyclonal, Goat)	R&D Systems	AF2085	1:125

Supplementary Table 5. Primers and UPL probes used for RT-qPCR.

Gene	Forward	Reverse	Probe
Alx4	GACACACTACCCTGATGTGTATGC	TCCCTCTTTCGCCACTTG	32
Cdx2	CACCATCAGGAGGAAAAGTGA	CTGCGGTTCTGAAACCAAAT	34
Foxc1	GCTTTCCTGCTCATTCGTCTT	AAATATCTTACAGGTGAGAGGCAAG	34
Foxc2	CGGCTAGGACTGGACAACTC	CTGACAGCTCGCATTGCTC	95
Foxd1	GACTCTGCACCAAGGGACA	TTCCTAACAATTGGAAATCCTAGC	47
Lef1	TCCTGAAATCCCCACCTTC	ACCCGTGATGGGATAAACAG	94
Meox2	CTAGACCTCACTGAAAGACAGGTG	TTGCTGTCCCCCTTTGAC	2
Msgn1	AATTACCTGCCGCCTGTCT	TGAGTGTCTGGATCTTGGTGA	106
Myf5	CTGCTCTGAGCCCACCAG	GACAGGGCTGTTACATTCAGG	10
Nkx1-2	CCAATCGGGTCACAGGAG	CGCATCCTCAGCTTCCTC	13
Nkx3-1	CGACTGAACCCGAGTCTGAT	AATCACCTGAGTGTGAGAGAAGG	5
Nkx3-2	GAGTAGGCTCCGGAAAAGC	TGTAGGGCTTCGCTCCTG	103
Pax1	CGGACGTTTATGGAGCAAAC	TCTTCCATCTTGGGGGAGTA	81
Pax3	GCGAGAAAAAGGCTAAACACA	CGGAGCCTTCATCTGACTG	110
Pax7	TACCAGCTGCCGGACTCTAC	CTGGGGCCTGTGTACTGTG	77
Pax9	AGCAGGAAGCCAAGTACGG	TGGATGCTGAGACGAAACTG	33
Sdha	TGTTCAGTTCCACCCCACA	TCTCCACGACACCCTTCTG	71
Sox2	GGCAGAGAAGAGAGTGTTTGC	TCTTCTTTCTCCCAGCCCTA	34
Sox9	GTACCCGCATCTGCACAAC	CTCCTCCACGAAGGGTCTCT	66
Tbp	GGGGAGCTGTGATGTGAAGT	CCAGGAAATAATTCTGGCTCA	97
T-Bra	CAGCCCACCTACTGGCTCTA	GAGCCTGGGGTGATGGTA	100
Tbx6	TGACTGATACTCGGCAAGCA	CGCTGTGGGGACAGAGAT	9
Uncx4.1	GCACCCGCACCAACTTTA	GCGATTTTGGAACCAGACC	25
Wnt3a	CTTAGTGCTCTGCAGCCTGA	GAGTGCTCAGAGAGGAGTACTGG	76
