# Supplementary material for: A TALE/HOX code unlocks WNT signalling response towards paraxial mesoderm
Source: Nat Commun. 2021 Aug 26;12:5136. doi: 10.1038/s41467-021-25370-4 (PMC8390530; doi:10.1038/s41467-021-25370-4)
Supplement: Supplementary file 11 — Description of additional supplementary files [file 41467_2021_25370_MOESM11_ESM.docx]

Description of additional supplementary files

Title: Supplementary Data 1.

Description: List of dissected embryonic tailbuds analysed by scRNA-seq, indicating genotype and number of cells sequenced for each embryo.

Title: Supplementary Data 2.

Description: ScRNA-seq of E8.5/E9.0 control and *Pbx* mutant embryonic tailbuds. Top100 enriched genes in each of the 13 clusters identified in E8.5/E9.0 control and *Pbx* mutant tailbuds. NMPs neuromesodermal progenitors, pNT pre-neural tube, MPCs/pPSM mesodermal progenitor cells/posterior pre-somitic mesoderm, aPSM anterior pre-somitic mesoderm, IM intermediate mesoderm, LPM lateral plate mesoderm, Alnt allantois, Endth endothelial cells, Bld blood, Endo endoderm, Spp splanchnopleura, Noto notochord, str stressed cells. Differential expression testing was performed with Seurat (v3.1)^70,71^ using a Wilcoxon Rank Sum test (FindAllMarkers function with default parameters). p_val, *P*-value not adjusted for multiple test correction; avg_logFC, average log2 fold change; pct.1, percentage of cells where the gene is detected in the cluster; pct.2, percentage of cells where the gene is detected on average in the other clusters; p_val_adj, adjusted *P*-value, based on Bonferroni correction using all genes in the dataset; gene, Ensembl gene ID.

Title: Supplementary Data 3.

Description: ScRNA-seq of WT EpiSCs differentiating to PSM at different time-points. Top100 enriched genes in each of the 7 clusters identified in WT EpiSCs differentiated in vitro to PSM and harvested at different time-points. EpiSCs epiblast stem cells, NMPs neuromesodermal progenitors, PSM pre-somitic mesoderm. Differential expression testing was performed with Seurat (v3.1)^70,71^ using a Wilcoxon Rank Sum test (FindAllMarkers function with default parameters). p_val, *P*-value not adjusted for multiple test correction; avg_logFC, average log2 fold change; pct.1, percentage of cells where the gene is detected in the cluster; pct.2, percentage of cells where the gene is detected on average in the other clusters; p_val_adj, adjusted *P*-value, based on Bonferroni correction using all genes in the dataset; gene, Ensembl gene ID.

Title: Supplementary Data 4.

Description: List of PBX1 and PBX2 coregulated genes at 12 h and 24 h of in vitro PSM differentiation. List of downregulated and upregulated genes in *Pbx-DKO* cells (RNA-seq) associated with PBX1/PBX2-occupied regions at 12 h and 24 h of in vitro PSM differentiation (ChIP-seq).

Title: Supplementary Data 5.

Description: De novo motif analysis of PBX-bound regions along differentiation. Complete list of Homer de novo motif results of 200 bp summit regions in EpiSCs and at 6 h, 12 h, 24 h and 48 h of differentiation. The enrichment analysis was performed using a one-sided hypergeometric test and default settings (no adjustments for multiple testing) with Homer (v4.1)^84^.

Title: Supplementary Data 6.

Description: List of PBX1, PBX2 and LEF1 coregulated genes at 24 h of in vitro PSM differentiation. List of downregulated and upregulated genes in *Pbx-DKO* cells (RNA-seq) associated with PBX1/PBX2 and LEF1- occupied regions at 24 h of in vitro PSM differentiation (ChIP-seq).

Title: Supplementary Movie 1.

Description: Time-lapse video of WT EpiSCs differentiating to PSM. WT EpiSCs were differentiated to PSM in vitro in the IncuCyte S3 Live-Cell Analysis System (Essen Bioscience), and images were acquired every h for 3 days. Time-lapse movies with all acquired pictures were further recreated using iMovie (v10.1.14).

Title: Supplementary Movie 2.

Description: Time-lapse video of *Pbx-DKO* EpiSCs differentiating to PSM. *Pbx-DKO* EpiSCs were differentiated to PSM in vitro in the IncuCyte S3 Live-Cell Analysis System (Essen Bioscience), and images were acquired every h for 3 days. Time-lapse movies with all acquired pictures were further recreated using iMovie (v10.1.14).
